# Acetonic Extract of *Buxus sempervirens* Induces Cell Cycle Arrest, Apoptosis and Autophagy in Breast Cancer Cells

**DOI:** 10.1371/journal.pone.0024537

**Published:** 2011-09-15

**Authors:** Ouardia Ait-Mohamed, Valentine Battisti, Véronique Joliot, Lauriane Fritsch, Julien Pontis, Souhila Medjkane, Catherine Redeuilh, Aazdine Lamouri, Christine Fahy, Mohamed Rholam, Djebbar Atmani, Slimane Ait-Si-Ali

**Affiliations:** 1 Laboratoire de Biochimie Appliquée, Faculté des Sciences de la Nature et de la vie, Université de Béjaia, Béjaia, Algeria; 2 Laboratoire Epigénétique et Destin Cellulaire, UMR7216, Centre National de la Recherche Scientifique (CNRS), Université Paris Diderot Sorbonne Paris Cité, Paris, France; 3 Laboratoire ITODYS, UMR7086 CNRS, Université Paris Diderot Sorbonne Paris Cité, Paris, France; University of Medicine and Dentistry of New Jersey, United States of America

## Abstract

Plants are an invaluable source of potential new anti-cancer drugs. Here, we investigated the cytotoxic activity of the acetonic extract of *Buxus sempervirens* on five breast cancer cell lines, MCF7, MCF10CA1a and T47D, three aggressive triple positive breast cancer cell lines, and BT-20 and MDA-MB-435, which are triple negative breast cancer cell lines. As a control, MCF10A, a spontaneously immortalized but non-tumoral cell line has been used. The acetonic extract of *Buxus sempervirens* showed cytotoxic activity towards all the five studied breast cancer cell lines with an IC_50_ ranging from 7.74 µg/ml to 12.5 µg/ml. Most importantly, the plant extract was less toxic towards MCF10A with an IC_50_ of 19.24 µg/ml. Fluorescence-activated cell sorting (FACS) analysis showed that the plant extract induced cell death and cell cycle arrest in G0/G1 phase in MCF7, T47D, MCF10CA1a and BT-20 cell lines, concomitant to cyclin D1 downregulation. Application of MCF7 and MCF10CA1a respective IC_50_ did not show such effects on the control cell line MCF10A. Propidium iodide/Annexin V double staining revealed a pre-apoptotic cell population with extract-treated MCF10CA1a, T47D and BT-20 cells. Transmission electron microscopy analyses indicated the occurrence of autophagy in MCF7 and MCF10CA1a cell lines. Immunofluorescence and Western blot assays confirmed the processing of microtubule-associated protein LC3 in the treated cancer cells. Moreover, we have demonstrated the upregulation of Beclin-1 in these cell lines and downregulation of Survivin and p21. Also, Caspase-3 detection in treated BT-20 and T47D confirmed the occurrence of apoptosis in these cells. Our findings indicate that *Buxus sempervirens* extract exhibit promising anti-cancer activity by triggering both autophagic cell death and apoptosis, suggesting that this plant may contain potential anti-cancer agents for single or combinatory cancer therapy against breast cancer.

## Introduction

Breast cancer, a major worldwide health issue, is considered as the most common malignancy and the most common cause of cancer-related death in Western countries [1]. Standard cancer therapy generally combines surgery, multi-therapeutic agents and ionizing radiation [2]. These anticancer agents induce cell cycle arrest and/or cell death by apoptotic or non-apoptotic mechanisms including necrosis, senescence, autophagy and mitotic catastrophe [3],[4].

Major issues concerning conventional anticancer chemotherapy are the occurrence of side effects induced by the non-specific targeting of both normal and cancer cells [5], [6], and the emergence of drug-resistant cancer cells [7]. Based on this, there has been growing interest in the use of naturally occurring molecules with chemo-preventive and chemotherapeutic properties in cancer treatment [8]–[12]. Natural products will thus continue to play major role as active substances, model molecules for the discovery and validation of drug targets [13], [14]. Among natural sources, plants have played an important role as a source of effective anticancer agents [15]–[17]. Four examples are well known: Taxol® from *Taxus brevifolia* L., vinca alkaloids from *Catharanthus roseus* G. Don, camptothecin from *Camptotheca acuminata*, Decne and podophyllotoxin from *Podophyllum peltuturn* L. [18], [19].

In folk medicine, *Buxus sempervirens* L. is used to treat rheumatism, arthritis, bile duct infections, diarrhea, fever and skin ulceration. Studies highlighted the unique feature of the genus *Buxus* regarding the presence of steroidal alkaloids (more than 200) [20]–[23]. The latter are known for exhibiting promising biological activities including anti-acetylcholine esterase [24]–[27], cytotoxic [28] and immunosuppressive activities [29]. Nevertheless, to our knowledge, no anticancer activity of *Buxus sempervirens* L. extracts has been yet described.

Based on folk medicine, we investigated here the cytotoxic effect of the acetonic extract of *Buxus sempervirens* L. against five breast cancer cell lines: MCF7, MCF10CA1a, T47D, BT-20 and MDA-MB-435 or the spontaneously immortalized cell line MCF10A as a control. Our results showed that the *Buxus* extract has specific cytotoxic effects toward cancer cell lines by mainly inducing a decrease in cyclin D1. Interestingly, the extract induced autophagic cell death and apoptosis in breast cancer cells tested and a caspase 3-independent apoptosis cell death in the aggressive MCF10CA1a cells.

## Results

### *Buxus* acetonic extracts exhibit cytotoxic properties and induce phenotype modifications in breast cancer cells

**Figure 1:**
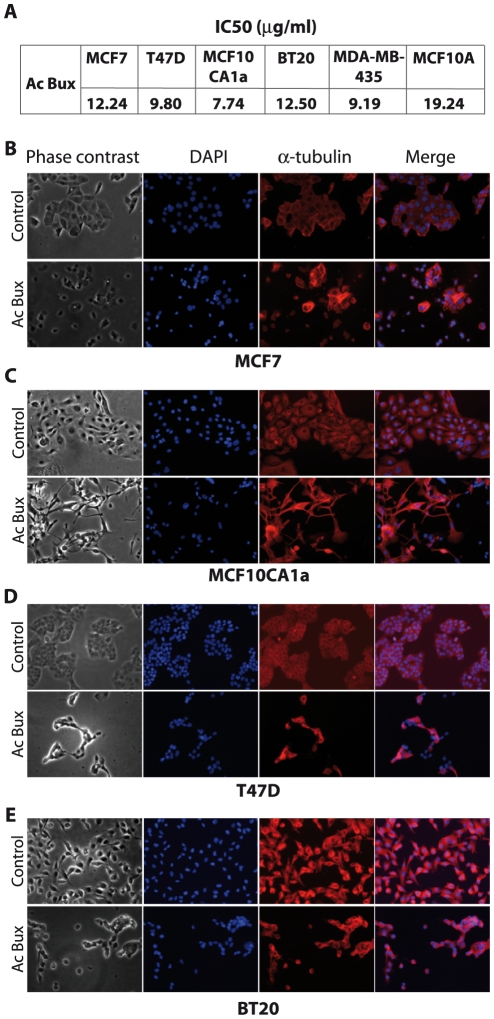
Cytotoxic effects of the acetonic extract of *Buxus sempervirens* L. towards breast cancer MCF7 and MCF10CA1a cells. **A.** IC_50_ determined by the dose-response curves obtained by the MTT assay. **B. C. D.** and **E.** Different cell shapes exhibited by MCF7, MCF10CA1a, T47D, MDA-MB-435 and BT-20, respectively, treated with *Buxus* extract at their respective IC_50_ during 72 h. Left panel: phase contrast images; Right panel: anti-α-tubulin fluorescence staining. Control cells are treated with vehicle DMSO (magnification ×200). Ac Bux: acetonic *Buxus* extract.

In order to evaluate the cytotoxicity of the acetonic extract of *Buxus*, an MTT assay was monitored on five breast cancer cell lines. The MCF7, MCF10CA1a and T47D, which are aggressive triple positive breast cancer cells, and BT-20 and MDA-MB-435 that are triple negative breast cancer cells. The extract exhibited cytotoxic activity toward all cancer cell lines tested, displaying reduced IC_50_ (<20 µg/ml) (Figure 1A). Moreover, the IC_50_ obtained against the control cell line MCF10A was higher (IC_50_ = 19.24 µg/ml, Figure 1A). These results suggest a specific cytotoxic effect mainly against breast cancer cell lines.

In order to give a better understanding of the mechanisms of cytotoxicity in cancer cells, we decided to carry on experiments on aggressive triple positive cancer cells: MCF7, MCF10CA1a, T47D and the triple negative breast cancer cell line BT-20.

First, major phenotypic changes were noticed when cancer cell lines were incubated in the presence of *Buxus* extract. Hence, interestingly, the cancer cell lines treated with the same extract (corresponding IC_50_ during 72 h) displayed different apoptotic cell shapes regarding the apoptotic volume decrease (AVD) (Figure 1B and 1C). To further test this, cytoskeleton staining (anti-α-tubulin) was applied. Treated MCF7, T47D and BT-20 cells exhibited a reduced round-shape cellular form before complete detachment from cell culture dish (Figure 1B, 1D and 1E), while MCF10CA1a cells showed a distinct and severe shrinkage (Figure 1C). These specific shapes are well known as the AVD due to massive efflux of K^+^ and Cl^−^ through their specific channels, leading to water escape from the cytoplasm, the latter being considered as a major hallmark of apoptotic cells [30], [31].

Finally, while DMSO-treated cells showed large nuclei with distinguishable nucleoli, we have noticed the transformation of nuclei into a unique pyknotic mass in dramatically-injured cells (Figure 1 B–E). On the other hand, normal MCF10A cells did not exhibit such dramatic phenotype changes. Together, our results suggest a cytotoxic activity of the *Buxus* extract regarding cancerous cells *via* apoptotic cell death.

### Acetonic extract of *Buxus* induces cell cycle arrest

**Figure 2:**
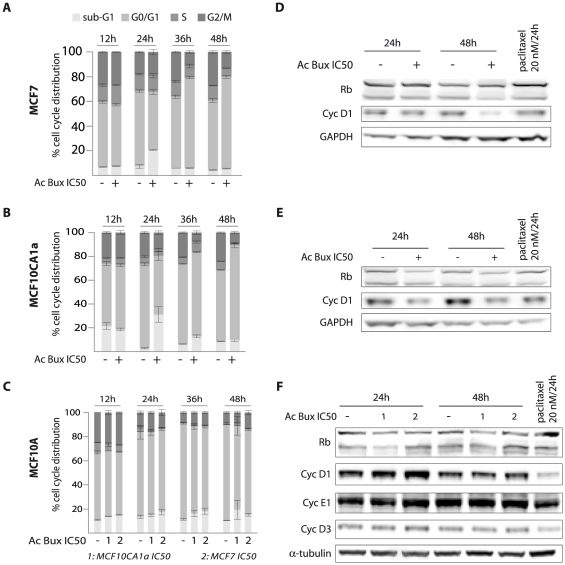
The acetonic extract of *Buxus* induces cell cycle arrest in MCF7 and MCF10CA1a breast cancer cell lines. **A.** MCF7 cells were incubated for increasing period intervals (12 h, 24 h, 36 h and 48 h) with their IC_50_ concentrations. The results represent means ± SEM of three experiments. **B.** MCF10CA1a cells were incubated for increasing period intervals (12 h, 24 h, 36 h and 48 h) with their IC_50_ concentration. The results represent means ± SEM of three experiments. **C.** MCF10A cells were incubated for the same period intervals (12 h, 24 h, 36 h and 48 h) with the IC_50_ of MCF7 and MCF10CA1a, respectively. The results represent means ± SEM of three independent experiments. **D.** Immunoblots of total cell extracts isolated from MCF7 treated or not with plant extract as indicated and probed with an anti-cyclin D1 antibody. GAPDH was used as a loading control. **E.** Immunoblots of total cell extracts isolated from MCF10CA1a treated or not with plant extract as indicated and probed with an anti-cyclin D1 antibody. GAPDH was used as a loading control. **F.** Immunoblots of total cell extracts isolated from MCF10A treated or not with plant extract (IC50s of MCF7 and MCF10CA1a concentrations) as indicated and probed with an anti-cyclin D1 antibody. α-tubulin was used as a loading control. Ac Bux: acetonic *Buxus* extract.

We studied the effect of the *Buxus* acetonic extract on the cell cycle of the studied breast cell lines. After 24 h incubation with the extract, stability is generally noticed in all cell cycle sub-populations of the control cell line MCF10A cells, with a slight increase in sub-G1 population observed with both concentrations applied (Figure 2C). We have also noticed a little decrease in the S-phase sub-population (Figure 2C). Interestingly, the IC_50_ were capable of triggering cell death of both cancerous cell lines. Thus, after 24 h of treatment, the sub-G1 sub-population sharply increased from 2.82% to 30.30% and from 7.31% to 20.64% for MCF10CA1a and MCF7, respectively (Figure 2A, Figure S1, S2). Concomitantly, there is a decrease in G0/G1 and S-phase sub-populations, mainly for MCF10CA1a cells from 69.59% to 48.05% and from 6.30% to 4.80%, respectively (Figure 2B). At 48 h, there is a significant increase in G0/G1 sub-population to the detriment of S and G2/M sub-populations (Figure 2A and 2B).

Finally, we have noticed in all cancer cell lines tested that a maximum of sub-G1 cell population is reached 24 h post-treatment, followed by a reduction (Figure 2A and 2B for MCF7 and MCF10CA1a, respectively). Concerning T47D and BT-20 cells, despite the observation of numerous floating dead cells, no major changes are illustrated in Sub-G1 sub-populations (Figure S3A and S3D). This could be due to the loss of the severely-damaged cells during washing steps. It is indeed established that the content of DNA remaining in apoptotic cells for cytometric analysis vary markedly depending on the extent of DNA degradation and cell washing steps [32]. Concerning MCF7 and MCF10CA1a, striking results were also noticed regarding the concentrations used: with high concentrations (2 times the IC_50_), there is an increase in sub-G1 population, while with low concentrations there is a decrease in S and G2/M phases (Figure S1A and S2A).

Concerning cell cycle markers, all cancer cells tested treated with IC_50_ during 24 h and 48 h showed a noticeable decrease in cyclin D1 expression (Figure 2D and 2E, and Figure S3 B–C and E–F). No major changes in the expression of Rb were noticed in treated cells, we have noticed a slight decrease in hypo-phosphorylated Rb protein levels 48 h after treatment (Figure 2D and 2E). Nonetheless, the IC_50_ of MCF7 and MCF10CA1a applied to MCF10A showed neither of the above effects (Figure 2C and 2F). These results indicate that the failure of tested breast cancer cells to enter S phase is due to a decrease in cyclin D1 induced by the *Buxus* acetonic extract.

### *Buxus* acetonic extract induces autophagy in breast cancer cells

**Figure 3:**
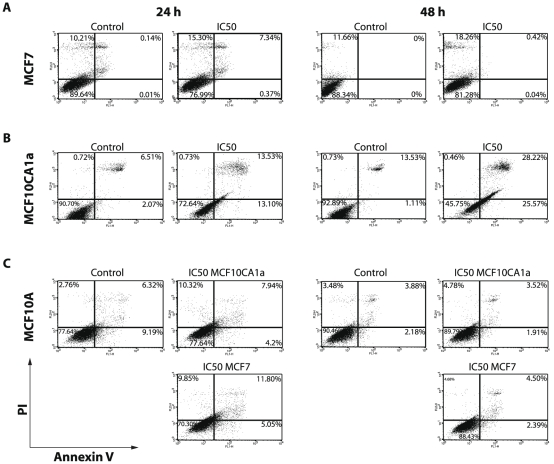
*Buxus* extract induces autophagy in cancer cells as evidenced by PI/Annexin V double staining and FACS analysis. **A–B.** PI/Annexin V double staining of untreated and treated MCF7 (A) and MCF10CA1a (B) cells with IC_50_ concentration for 24 h and 48 h. **C.** FACS analysis with PI/Annexin V double staining of MCF10A cell line (control cell line) treated with MCF7 and MCF10CA1a IC_50_ respective *Buxus* extract concentrations for 24 h and 48 h.

We have next investigated the role of *Buxus* acetonic extract in cell death. To this end, cells were collected after 24 h and 48 h treatment with respective IC_50_, double-stained with PI and Annexin V-FITC and analyzed by FACS (Figure 3 and Figure S4). The kinetic of cell interaction with Annexin V revealed that the extract acts very fast (not shown). Interestingly, there is a discrepancy in the behavior of the breast cancer cell lines. Indeed, while with MCF10CA1a, T47D and BT-20 we revealed a pre-apoptotic sub-population (PI^−^/Annexin V^+^) (13.10% versus 25.57% after 24 h and 48 h of treatment, respectively for MCF10CA1a as an example), that latter shifted to a late apoptotic and/or a necrotic sub-population (PI^+^/Annexin V^−^ quadrant) (Figure 3B, Figure S4 A–B). However, with MCF7 cell line, we noticed that the cell population shifted directly to PI^+^ quadrants (dead cells) without transition by the PI^−^/AnnexinV^+^ (Figure 3A), even with reduced time contact kinetics (one hour intervals, data not shown). These findings suggested that the process of death induced by *Buxus* acetonic extract differs in the cancer cell lines; MCF10CA1a, BT-20 and T47D cells die *via* apoptosis pathway, while MCF7 cell death seemed to rely mainly on autophagy.

As previously seen with PI staining, reduced cell death is observed with MCF10A, even after 48 h of treatment, confirming the specific effect on cancerous cell lines. Paradoxically, a more lethal action is noticed after 24 h of incubation compared to 48 h (Figure 3C).

**Figure 4:**
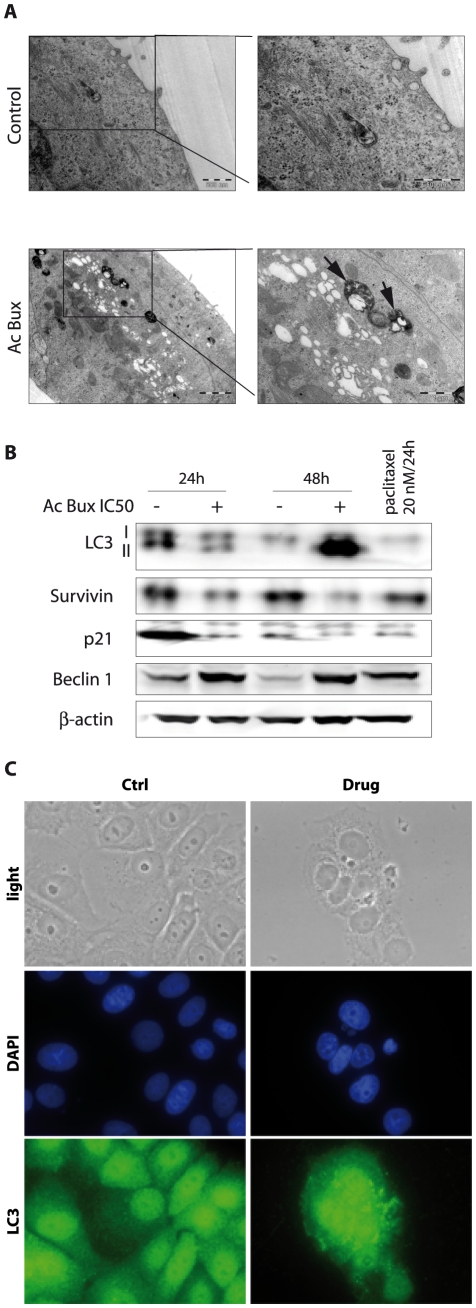
Acetonic extract of *Buxus* induces autophagy in MCF7 cell line. **A.** Transmission electron microscopy pictures of untreated and *Buxus* extract-treated MCF7 cells with IC_50_ concentration for 72 h. Black arrows show degradative autophagic vesicles. White arrows show lucent electron vesicles. **B.** Immunoblots of total cell extracts isolated from MCF7 (treated and untreated, as indicated) probed with different antibodies demonstrating the occurrence of autophagy. β-actin has been used as a loading control. **C.** Immunofluorescence targeting LC3 obtained with untreated and *Buxus* extract-treated MCF7 cells (IC_50_, 72 h). Magnification ×400. Ac Bux: acetonic *Buxus* extract.

According to pictures obtained with transmission electronic microscopy, untreated MCF7 cells displayed normal characteristics with, however, the presence of some auto-lysosomes/auto-phagosomes in cell cytoplasm (Figure 4A), suggesting that even in normal growth conditions, MCF7 cells proceed to some controlled autophagy. Nevertheless, treated MCF7 cells with the *Buxus* acetonic extract (IC_50_ during 72 h) showed abundant auto-lysosomes/auto-phagosomes dispersed in the cytoplasm (Figure 4A). Hence, in the presence of the plant extract, the phenomenon is dramatically increased, leading to cell death without any damage to mitochondria and cytoplasmic membrane. These observations suggested that MCF7 death is due to autophagy rather than apoptosis. This is in agreement with previous reports showing that MCF7 cells do not undergo apoptosis after treatment with numerous apoptosis stimuli, including Tamoxifen [33], or injection of supra-physiological amounts of cytochrome C [34].

**Figure 5:**
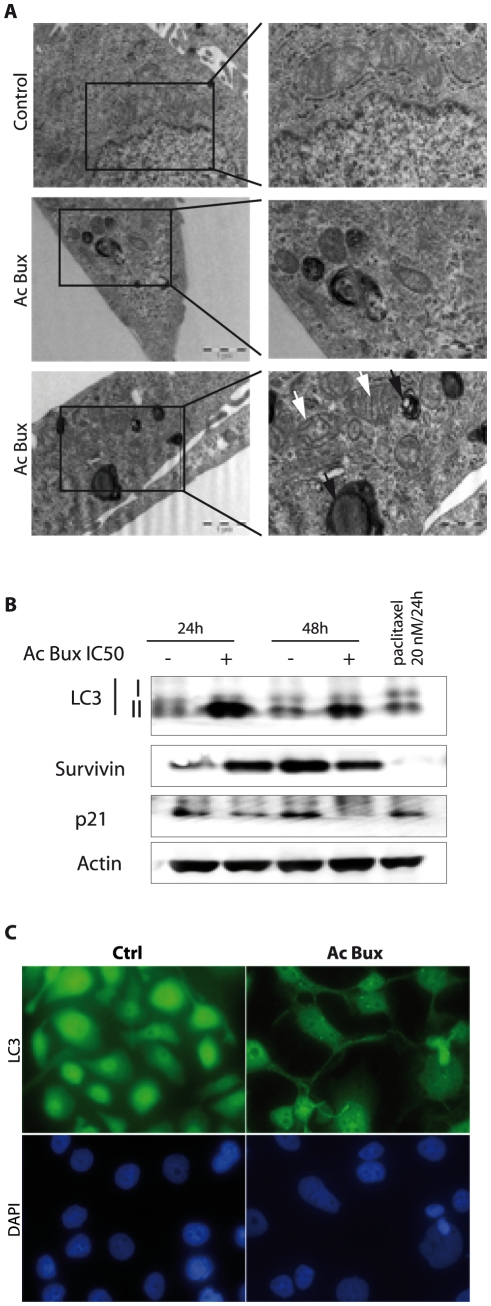
Acetonic extract of *Buxus* induces autophagy in MCF10CA1a cell line. **A.** Transmission electron microscopy pictures of untreated and *Buxus* extract-treated MCF10CA1a cells (IC_50_, 72 h). Black arrows show degradative autophagic vesicles. White arrows show damaged mitochondria. **B.** Immunoblots of total cell extracts isolated from MCF10CA1a (treated and untreated, as indicated) probed with different antibodies demonstrating the occurrence of autophagy. β-actin has been used as a loading control. **C.** Immunofluorescence targeting LC3 obtained with untreated and treated MCF10CA1a cells (IC_50_, 72 h). Magnification ×400. Ac Bux: acetonic *Buxus* extract.

Concerning MCF10CA1a cells, pictures taken after IC_50_ treatment during 72 h, provided several hallmarks of apoptosis and autophagy (Figure 5A). We noticed the presence of initial autophagic vacuoles and degradative autophagic vacuoles, peri-nuclear localization of mitochondria, and most importantly, some of them were damaged.

To carry on our investigation concerning autophagy we studied a main autophagy marker, the Microtubule associated Light Chain 3 or LC3 protein. LC3 is the mammalian homolog of the yeast Apg8p protein, essential for amino acid starvation-induced autophagy [35], [36]. LC3 is present in two forms in cells: LC3-I is the cytoplasmic form, which is processed into a lipidic LC3-II form, associated with the auto-phagosome membrane [35], [36]. Therefore, we compared the LC3 distribution in *Buxus* acetonic extract-treated and untreated cells (Figures 4B–C, 5B–5C, Figure S5). In DMSO-treated cells, we noticed a homogeneous cytoplasmic distribution of unprocessed LC3-I, while in plant extract-treated cells (IC_50_/72 h), many foci are depicted, corresponding to lipidic transformed LC3-II, mainly around nuclei (Figures 4C for MCF7, 5C for MCF10CA1a, Figure S5 A for T47D and C for BT-20). This specific signal corresponds to the auto-phagosome trans-membrane processed version of LC3. These results are in agreement with images taken with transmission electron microscopy (Figures 4A and 4B for MCF7 and MCF10CA1a respectively), where we noticed accumulation of late auto-phagosomes mainly around cell nuclei. In the case of MCF10CA1a cells, the foci pattern of LC3-II was difficult to confirm since there was very little cytoplasm around nuclei (Figure 5C).

Concerning immunoblots, the presence of LC3-II in untreated (24 h) MCF7 cells, demonstrated the occurrence of controlled-autophagy in normal cells, as already seen with transmission electron microscopy (Figure 4A). For MCF10CA1a aggressive cells, we found a decrease in LC3-II in *Buxus* acetonic extract-treated cells (Figure 5C). This is probably because LC3-II is present both on inner and outer auto-phagosome membranes, with the former being degraded inside auto-lysosomes, whereas LC3 on the outer membrane is deconjugated by Atg4 (Autophagy related gene 4) and returns to the cytosol [35]. Finally, concerning the control cell line MCF10A, a faint LC3-II signal is detected when the cells were treated with the IC_50_ of MCF7 (Figure S6). Immunoblots of total cell extracts from treated and non-treated T47D and BT-20 confirmed also autophagy processing since we have noticed the processed form of LC3 (LC3 II, 24 h and 48 h after treatment) (Figure S5 B and D for T47D and BT-20, respectively).

### Acetonic *Buxus* extract induces caspase 3-independent apoptosis in MCF10C1a

**Figure 6:**
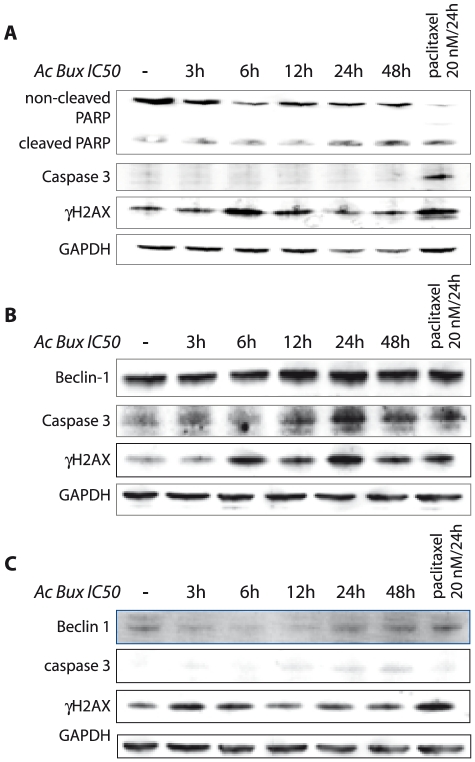
*Buxus* extract induces apoptosis in MCF10CA1a, T47D and BT-20 breast cancer cell lines. **A.** Immunoblots of total extracts from MCF10CA1a, revealing the presence of the cleaved from of PARP and γH2AX, hall marks of apoptosis, at the same time, the blot reveals the absence of active caspase 3, demonstrating the occurrence of apoptosis without caspase 3 activation. **B.** and **C.** Immunoblots of total extracts from T47D and BT-20, respectively, revealing the presence of the cleaved from of caspase 3 and γH2AX, hallmarks of apoptosis, demonstrating the occurrence of apoptosis and an up-regulation of Beclin-1, proving the occurrence of autophagy at the same time. GAPDH was used as a loading control. Ac Bux: acetonic *Buxus* extract.

In order to get more insights on the pattern of cell death, mainly in MCF10CA1a, we studied the activation of several additional markers related to apoptosis by immunoblot (Figure 6A). Pro-caspase 3 is undetectable in MCF-7 cells due to a 47-bp deletion within exon 3 of the procaspase-3 gene that alters the reading frame of the message, resulting in an unstable truncated polypeptide [34], [37]. According to that, activated caspase 3 was assessed in MCF10CA1a (Figure 6A), as well as in the control cell line MCF10A (Figure S6). Surprisingly, active caspase 3 was absent after treatment with the plant extract, even with reduced incubation times (Figure 6A). This result is in contradiction with our previous finding concerning Annexin V staining; the aggressive cell line MCF10CA1a displayed PI^−^/Annexin V^+^ pattern after plant treatment, illustrating an apoptotic cell death concomitant to autophagy. Taken together, these results indicate that MCF10CA1a death can be related only to autophagy, triggered by metabolic stress created by damaged mitochondria that caused an energy-deprivation state, or the autophagy is coupled to an apoptosis cell death independent of caspase 3 activation, since we noticed occurrence of DNA damages related apoptosis (presence of cleaved PARP and γH2AX, Figure 6A).

As the cells displayed a G1-phase arrest, we were interested in testing levels of p21, a potent cell cycle inhibitor through inactivation of G1-phase cyclin/CDK complexes. Surprisingly, we have found a decrease in p21 levels in cancer cell lines tested (Figures 4B and 5B, Figure S5 B and D). In addition, the cells showed reduced levels of Survivin after plant extract treatment. In the control cell line MCF10A, Survivin was detected at 24 h but no effect on its levels is noticed after plant extract treatment. At 48 h, the level of Survivin is undetectable along with Cyclin A2 (Figure S6), this can be explained by the fact that the cell line did not undergo mitosis and can hence explain the disappearance of Survivin.

It is known that the up-regulation of Survivin expression in cancer cells is independent of the cell cycle, suggesting an increase of its anti-apoptotic role compared to normal cells, in which its mitotic regulation functions may be predominant.

Beclin 1 is a 60-kDa protein that plays a critical role in the formation of auto-phagosomes in mammalian cells [38], [39]. 40% of human breast carcinoma cell lines exhibit deletions of one or more alleles of *beclin 1* gene [40]. This decreased expression of Beclin 1 suggests that specific molecular alterations in autophagy pathways may contribute to tumorigenesis [41]. As illustrated in Figure 4, 6B and 6C, an increase in Beclin 1 levels was noticed in treated MCF7, T47D and BT-20, respectively, demonstrating that the plant extract triggers autophagic cell death.

### Acetonic *Buxus* extract induces apoptosis in T47D and BT-20

Since we have noticed the presence of pre-apoptotic subpopulations in Annexin V-FITC stained cells, we decided to check the occurrence of apoptosis in these cell lines. As illustrated in Figure 6B and 6C for T47D and BT-20, respectively, after 3 h of treatment, there is occurrence of apoptosis since there is expression of certain apoptosis markers : caspase 3, γH2AX. In parallel, autophagy occurs in these cells, since there is a concomitant overexpression of Beclin-1 (Figure 6B and 6C).

## Discussion

In this study, we report cytotoxic effects of a plant extract – acetonic extract of *Buxus sempervirens* L. – on several breast cancer cell lines. Cytotoxic activities concerning *Buxus* species are scarce; although an interesting cytotoxic activity is reported for triterpenoid alkaloids isolated from *Buxus microphylla* L. against HepG2 [28]. According to our results, in breast cancer cell lines, the *Buxus* acetonic extract induced cell cycle arrest in G0/G1 phase and triggered cell death by increased sub-G1 cell population. The observed effects could be mediated by two sub-classes of cytotoxic molecules, a first class could act fast and require high concentration to induce cell death, and a second class plays a role in cell cycle arrest by preventing the G1-to-S transition. Alternatively, all these effects could be attributed to a single molecule. This conclusion arises from previous similar results described in the literature with Resveratrol [42]. Indeed, this phytoalexin stilben isolated from grapes, wine and nuts, induces cell cycle arrest at low concentrations and cell death through auto-phagocytosis process in ovarian cancer cells at high concentrations [42]. This is a very striking finding, since our preliminary results revealed the absence of Resveratrol in *Buxus* extracts. Also, it is worthy to notice that the plant was collected in an area characterized by unfavorable growth conditions (mountainous and semi-arid region) which are known to trigger the production of phytoalexin substances.

Our investigation concerning cell cycle arrest revealed also a highly sought characteristic. The *Buxus* acetonic extract is able to block cell cycle in G0/G1 through the decrease in cyclin D1. Cyclin D1 belongs to the family of three closely related D-type cyclins, D1, D2 and D3, which are redundant in all proliferating cell types. D-cyclins together drive cell-cycle progression by activating their cyclin-dependent kinase partners, CDK4 and CDK6, which leads to phosphorylation of the retinoblastoma protein (Rb), and in turn to the advance through the G1 phase of the cell cycle [43], [44]. Cyclin D1 is over-expressed in most breast tumor cell lines through over-expression and/or amplification at its genomic locus, 11q13. This feature has been shown to play a key role in tumorigenesis and confers bad prognosis in breast cancer [45]–[47]. Moreover, the cell cycle arrest observed cannot be the result of CDK inhibitors activation as shown by decreased levels of p21 and p27 (data not shown). Rather, the effect relies on a direct decrease in cyclin D1, strongly suggesting that inhibition of cyclin D1 by *Buxus* extract could be a good tool to improve prognosis in breast cancer.

Another interesting feature concerns the concomitant occurrence of the two programmed cell deaths, apoptosis and autophagy, in several breast cancer cells including triple positive and triple negative ones, since our results have shown markers related to both of them. Transmission electron microscopy analyses showed marked differences in localization and shapes of mitochondria in *Buxus* extract-treated MCF10CA1a cells. The cellular distribution of mitochondria is deeply affected during apoptosis. Mitochondria are normally dispersed throughout the entire cell; however, during apoptosis triggered by tumor necrosis factor (TNF), there is a peri-nuclear clustering of mitochondria is caused by an impaired activity of the molecular motor kinesin [48]. Also, the loss of integrity of the mitochondria outer membrane is a very important hallmark of apoptosis. Referred as MOMP (Mitochondrial Outer Membrane Permeabilization), it leads to the release of proteins normally found in the space between the inner and outer mitochondrial membranes, such as cytochrome C and AIF (Apoptosis Inducing Factor) [49]. It is well established that the release of these molecules initiates apoptosis. Cytochrome C binds to APAF-1 (apoptotic protease activating factor–1). In the presence of ATP, APAF-1 is allowed to oligomerization and forms the “apoptosome” which, in turn, activates Caspase 9 by dimerization. The active Caspase 9 activates executor caspases (Caspase 3 and 7) and this orchestrates apoptosis through the cleavage of key substrates within the cell [50]. Also, AIF has a direct effect on isolated nuclei, triggering chromatin condensation as well as large-scale chromatin fragmentation [51].

There is more and more evidence that autophagy is a mechanism of cell survival following plethora of extra-cellular and intra-cellular stimuli. Numerous studies have demonstrated that proceeding to autophagy allows cancer cells to escape cell death [52]–[55]. Nevertheless, the autophagy is highly contextual, it can exert both cyto-protective and death-promoting effects. Indeed, the effect of autophagy may vary dependent on the type of cancer, individual characteristics of cancer cells, microenvironments, and therapeutic treatment [52]. Nonetheless, it is clearly assumed that induction of autophagy to high levels leads to autophagic cell death [56], [57].

Interestingly, the *Buxus* extract induced a decrease in p21 levels, which could be related to its involvement as an anti-apoptotic protein. This is exemplified by preventing apoptosis by protecting the N-terminal moiety of Caspase 3 preventing its activating proteolysis [58]. Lately, p21 has been reported to play a crucial role in autophagy [59]; although, the entire mechanism is not fully understood. Wild-type MEF (Mouse Embryonic Fibroblasts) undergo apoptosis upon C2-ceramide treatment, and *p21* ^−/−^ MEF undergo autophagy rather than apoptosis upon the same death stimulus. p21 triggers apoptosis by inhibiting the autophagic pathway through the suppression of the stability of autophagy-related proteins in MEF [59]. Hence, decreased levels of p21 observed in the cells treated with the plant extract can trigger cell death by autophagy.

By decreasing levels of p21, the *Buxus* extract seems to contain molecules that inhibit cytosolic p21 and trigger cell death. It has been already shown that targeting p21 (with an anti-sense oligodeoxynucleotide) attenuated the growth of Met-1 tumors in nude mice [60]. Finally, our data demonstrated that the *Buxus* extract also decreases levels of Survivin, a 16.5 kDa protein that belongs to the IAP family (Inhibitor of Apoptosis proteins) [61], which plays a key role in mitotic spindle formation [62]. However, two general considerations make Survivin an attractive therapeutic target in cancer: it is selectively expressed in tumor cells and it is required for their viability [63], [64]. In cancer cells, Survivin correlates with unfavorable prognosis, resistance to therapy, and accelerated rates of recurrences [65].

In light of our results, we can conclude that *Buxus* sempervirens extract targets many proteins widespread in cancer cells cytoplasm, leading to cell cycle arrest and autophagy. There is however a crosstalk between apoptosis and autophagy, which determines cell fate, but the molecular mechanism is not fully understood. Previous data suggested that the removal or functional inhibition of essential proteins from the apoptotic machinery can switch a cellular stress response from the apoptotic default pathway to a state of massively increased autophagy. However, apoptosis develops only when autophagy is inhibited [66]. In our case, mechanisms of the concomitant occurrence of autophagy and apoptosis are unclear. A possible explanation for the autophagy observed in MCF10CA1a cells can be the presence of Ha-Ras. This aggressive cell line was obtained by transfecting MCF10A with this oncogene. It has been lately shown that the presence of this signature leads to the occurrence of autophagy [67].

### Conclusion

Nowadays, it is accepted that the major problem with conventional chemotherapy lies in the doses used: low doses have no effect on cancer cells and too high doses induce deleterious side effects. Thus, the presence of a “sensitizer” that can force cells to undergo apoptosis even with mild DNA-damaging agents would greatly enhance the efficacy and limit side effects of conventional chemotherapy drugs [68]. Hence, targeting p21 and Survivin can be a good adjuvant therapy to improve cell death in accompaniment to other conventional drugs [69], [70]. *Buxus* extract probably contains molecules that inhibit p21 and Survivin and thus can be used in addition to commonly used drugs to trigger cell death.

Another important feature concerning *Buxus* extract is its capacity to target the cell cycle which is very promising in cancer chemotherapy. Agents that induce cell cycle arrest are increasingly used in combination with traditional cytotoxic drugs to overcome cell cycle–mediated drug resistance and to improve cytotoxic efficacy. Among them, Flavopiridol has been shown to directly inhibit many CDK proteins [71].

Taken together, our data suggest that *Buxus sempervirens* extract can induce cell death not only via apoptosis, but also by autophagy. This is very promising, since it indicates that the *Buxus* extract may contain molecules that can be potentially used in apoptosis-resistant cells. Also, it exhibited increased toxicity towards cancer cell lines, including triple negative breast cancer cells. Moreover, it induced cell cycle arrest, depletion of cell energy, leading to cell death. Finally, *Buxus* deserves further investigation to understand the potential use of its molecules in therapeutic application for cancer treatment.

## Materials and Methods

### Plant extract preparation

*Buxus sempervirens* L. (Buxaceae) was collected from remote places around the province of Béjaia (with the kind permission of the Parc National de Dujurdjura authorities, Northeastern region of Algeria) in March 2008. Plant parts (leaves and flowers) used in this study were chosen on the basis of their use in Algerian ancestral medicine.

Powdered material (2 g) was macerated in pure acetone (200 ml) during 24 h, at room temperature with light stirring (50 rpm), and then filtered using 0.22 µm filters (Millipore). The flow-through material was evaporated to dryness under reduced pressure and the solid extract was reconstituted in DMSO solvent (100 µg/µl stock solution) before storage at −20°C.

### Cell culture

MCF7 cells (HTB-22, ATCC) were grown in Dulbecco's Modified Eagle Medium (DMEM), 4.5 g/l of glucose, supplemented with 5% fetal calf serum, 100 U/mL of penicillin (PAA), and 100 µg/mL of streptomycin (PAA). MCF10A cells (CRL-10317, ATCC) were cultured in DMEM/F-12 medium (PAA, Carlsbad, CA) supplemented with 10 µg/mL of human insulin (Sigma, St. Louis, MO), 20 ng/mL of epidermal growth factor (Sigma, St. Louis, MO), 0.5 µg/mL of hydrocortisone (Sigma, St. Louis, MO), 5% horse serum (Invitrogen), 100 U/mL of penicillin (PAA) and 100 µg/mL of streptomycin (PAA). T47D (HTB-133, ATCC), a generous gift from Dr Yegor Vassetzky were grown in DMEM, 4.5 g/l of glucose, supplemented with 10% horse serum, 100 U/mL of penicillin (PAA), and 100 µg/mL of streptomycin (PAA). BT-20 (HTB-20, ATCC) and MDA-MB-435 cells (HTB-129, ATCC) were cultured in DMEM, 4.5 g/l of glucose, supplemented with 10% fetal calf serum, 100 U/mL of penicillin (PAA), and 100 µg/mL of streptomycin (PAA). MCF10CA1a cells [72] were cultured in DMEM/F-12 medium supplemented with 5% fetal calf serum (PAA), 100 U/mL of penicillin (PAA) and 100 µg/mL of streptomycin (PAA). All cited cells were cultured at 37°C in a humidified atmosphere and 5% CO_2_.

### Viability assay

Cell proliferation was determined using the Cell Titer Glo assay (Promega). Cells were seeded at a density of 3×10^3^ cells per well in 96-well plates and maintained 24 h for attachment and then treated with two-fold serial dilutions of the plant extract. After 72 h incubation, 20 µL of MTT reagent were added. The plates were incubated during 2 h and absorbance determined at 560 nm in Glomax Multi-detection System (Promega). Percentages of cell survival were calculated as follows: % cell survival = (absorbance of treated cells/ absorbance of cells with vehicle solvent)×100. The half inhibitory concentration (IC_50_) was calculated from the dose–response curve obtained by plotting the percentage of cell survival *versus* the concentration of plant extract used. All assays were performed three times in duplicate. During all experiments, DMSO dilutions of *Buxus* acetonic extract were adjusted in the culture media at a final concentration of 0.2% (v/v).

### FACS analysis, PI and PI/Annexin V staining

In order to determine the effect of plant extract on the cell cycle, FACS analysis was carried out. For propidium iodide (PI) staining, cells were seeded in 6-well plates at a density of 10^4^ cells/ml. After 24 h of attachment, cancer cells were treated with indicated plant extract concentrations for different time intervals. Floating and attached cells were harvested, washed in PBS, fixed in ice-cold ethanol (70% v/v) and stored at −20°C. For analysis, cells were washed in PBS and suspended in PI (25 mg/ml) in PBS with RNase A (200 µg/ml).

For PI/Annexin V double staining, treated cells were harvested and suspended in binding buffer (HEPES pH 7.4, CaCl_2_ 2.5 mM, NaCl 140 mM). Aliquots of cells were incubated for 15 mn with Annexin V FITC and PI (5 µg/mL) (Invitrogen).

During all FACS analyses, 10^5^ events for each sample were analyzed. Flow cytometry analyses were carried out on a FACScalibur system (BD Biosciences) followed by analysis using CellQuest Pro software (BD Biosciences).

### Ultra-structural study by transmission electronic microscopy

Treated (IC_50_/72 h) and control cells were fixed in buffered (0.1 M) sodium cacodylate, pH 7.4 and 2.5% glutaraldehyde solution for 2 h. After washing, the cells were post-fixed in 1% OsO_4_ solution for 1 h at room temperature, rinsed and dehydrated in an ethanol gradient (70% to 100%, 10 min for each bath). Absolute ethanol was replaced by 2,3 epoxy propylether and further by propylene oxide. Cells were infiltrated by epoxy resin (R1165, Agar scientific) mixed to propylene oxide (50%-50%) overnight, followed by three baths with pure epoxy resin. Samples were polymerized at 60°C during 18 h. Ultra-thin sections (80 nm) cut with an ultra-microtome (Leica UC6) were stained with uranyl acetate (20 min) and Reynolds lead citrate (2 min). Sections were observed at 80 kV, in a TEM Phillips Tecnai equipped with an Olympus Keenview CCD camera.

### Immunofluorescence

Cells were grown on Permanox slides during 24 h before *Buxus* acetonic extract treatment (IC_50_, 72 h). They were fixed with a paraformaldehyde solution (4%) and permeabilized with 0.1% Triton X-100 in PBS, before incubation with appropriate antibodies: α-tubulin (1/5000, Sigma), LC3 (1/200, Sigma) overnight at 4°C. After extensive washing, slides were incubated 1 h at room temperature with red fluorescent Alexa Fluor 568 dye-labeled anti-mouse IgG for α-tubulin and green-fluorescent Alexa Fluor 488 dye-labeled anti-rabbit for LC3. Coverslips were mounted in DAPI (4′,6-diamidinole-2-phenolindole) (Sigma Aldrich). Finally, cells were observed with a Leica DMI 6000 B microscope and images were treated with MetaMorph software.

### Western blot

Cell extracts were prepared in RIPA (50 mM Tris-HCl (pH 7.5), 150 mM NaCl, 1% NP40, 0.5% Na-deoxycholate, 0.1% SDS, 1 mM EDTA containing protease inhibitor mixture (Roche Applied Science). After sonication on a Bioruptor (Diagenode) at high frequency during 7.5 mn (1 mn of pause *versus* 15 sec of sonication), the soluble protein fraction was collected after centrifugation at 13 500 g during 10 mn. Protein concentration was determined BCA kit according to manufacturer's instructions (Pierce, Rockford, IL). 30 µg of proteins were subjected to SDS-PAGE in 4 to 12% gradient gels and separated proteins were transferred to nitrocellulose membrane (Invitrogen). Incubation with different antibodies was monitored overnight at 4°C. Membranes were incubated with the appropriate secondary antibody coupled to HRP (Horseradish Peroxidase), revealed using West Dura kit (Pierce, Rockford, USA) and ChemiSmart 5000 system (Vilber Lourmat).

### Antibodies

The antibodies against p21 (C-19, sc-397), cyclin A2 (C-19, sc-596), cyclin D1 (DCS-6, sc-20044), cyclin D3 (C-16, sc-182), cyclin E1 (E-4, sc-25303), Rb (C-15, sc-50) were from Santa Cruz Technologies. Anti-cleaved caspase 3 (Asp 175 9661) was purchased from Cell Signaling Technologies. Anti-PARP (33–3100) was from Zymed Inc, anti-LC3 (L8918). Antibodies against Beclin-1 (B6061), β-actin (T9026), α-tubulin (A5441) and GAPDH (G8795), normal mouse and normal rabbit IgG were from Sigma Aldrich. Anti-Survivin (Ab469) was from Abcam.

### Statement of Ethics

Experimental research reported in the manuscript must have been performed with the approval of the ethic committee of our Department following the French and European rules. No research on humans has been carried out.

## Supporting Information

Figure S1 **Dose effect of the acetonic extract of** ***Buxus*** **on MCF7 cells.** A. FACS analysis of treated MCF7 with increasing concentrations of *Buxus* extract. The results are the mean ± SEM of three experiments. The results demonstrate a dose effect with increased sub-G1 subpopulation upon plant extract treatment. B. Immunoblot analyses of total extract of treated MCF7 cells treated with increasing concentrations of *Buxus* showing the multiple targets of the extract at high concentrations. GAPDH was used as a loading control.
(EPS)

Figure S2 **Dose effect of the acetonic extract of** ***Buxus*** **on MCF10CA1a cells.** A. FACS analysis of treated MCF10CA1a with increasing concentrations of *Buxus* extract. The results represent means ± SEM of three experiments. The results demonstrate a dose effect with increased Sub-G1 subpopulation upon plant extract treatment. B. Immunoblot of total extract of treated MCF10CA1a cells treated with increasing concentrations of *Buxus* showing the multiple targets of the extract at high concentrations. GAPDH was used as a loading control.
(EPS)

Figure S3 **Treatment of T47D (an aggressive triple positive breast cancer cell line) and BT-20 (a triple negative brast cancer cell line) cells with Bux acetonic extracts resulted in the accumulation of cells in G0/G1 phase in a dose- and time-dependent fashion.** **A. and D.** T47D and BT-20, respectively, were treated in increasing concentrations of the plant extract (IC50/2, IC50 and 2× IC50) during 24 h and 48 h and resulted in an accumulation of cells in G0/G1 phase as demonstrated by FACS analyses. The results represent means ± SEM of three experiments. **B and E.** Western blots analysis of untreated and treated T47D and BT-20 cells, respectively, showing a decrease of cyclin D1 after their respective IC50 treatment with the plant extract during 24 h and 48 h. **C and F.** Western blot analysis of total cell extracts from untreated and treated T47D and BT-20 cells, respectively, with increasing concentrations of Ac Bux (IC50/2, IC50 and 2× IC50) during 48 h, illustrating a dose effect of Ac Bux on the several targeted proteins probed. Ac Bux: acetonic Buxus extract.
(EPS)

Figure S4 **Treatment of T47D and BT-20 cells with the plant extract resulted in the accumulation of apoptotic/ necrotic cells.** **A.** T47D cells were treated during 24 h and 48 h with the plant extract (IC_50_) and resulted in the accumulation of apoptotic/necrotic cells as illustrated with the Annexin V-FITC stained cells analyzed by FACS. **B.** Annexin V-FITC stained BT-20 cells showing that after 24 h of plant extract treatment (IC_50_), there is an emergence of pre-apoptotic cells that shift to apoptotic/ necrotic cell population after 48 h of treatment.
(EPS)

Figure S5 ***Buxus*** **extract treatment in T47D and BT-20 cells resulted in autophagy.** **A and C.** Immunofluorescence targeting LC3 obtained with untreated and treated T47D and BT-20 cells, respectively, (IC_50_, 72 h), showing the punctuated staining of the processed form LC3 II. Magnification ×400. Ac Bux: acetonic *Buxus* extract. **B and D.** Immunoblots of total cell extracts of untreated and treated T47D and BT-20 cells, respectively, (IC_50_ during 24 h and 48 h) demonstrating the occurrence of the processed form of LC3 and the decrease of p21 levels.
(EPS)

Figure S6 **Immunoblot analysis of total cell extracts isolated from MCF10A demonstrating the absence of the processed LC3II and caspase 3 in treated cells.** Only a small band related to LC3II is present when cells were treated with MCF7-IC50. α-tubulin was used as a loading control.
(EPS)
